# Research Project Grants in Gynecology and Their Influence on Practice Guidelines

**DOI:** 10.7759/cureus.85730

**Published:** 2025-06-10

**Authors:** Mateo G Leon, Ellen H Crowe, Han-Yang Chen, Stephen M Wagner, Katherine Lambert, Shelby Irwin, Suneet P Chauhan

**Affiliations:** 1 Division of Advanced Minimally Invasive Gynecologic Surgery, Department of Obstetrics, Gynecology, and Reproductive Sciences, McGovern Medical School, The University of Texas Health Science Center at Houston, Houston, USA; 2 Division of Maternal Fetal Medicine, Department of Obstetrics, Gynecology, and Reproductive Sciences, McGovern Medical School, The University of Texas Health Science Center at Houston, Houston, USA; 3 Department of Obstetrics and Gynecology, Beth Israel Deaconess Medical Center, Harvard Medical School, Boston, USA; 4 Department of Obstetrics and Gynecology, University of Texas Medical Branch, Galveston, USA; 5 Department of Obstetrics and Gynecology, University of South Florida Health, Tampa, USA; 6 Department of Obstetrics and Gynecology, Delaware Center for Maternal &amp; Fetal Medicine for Christiana Care, Newark, USA

**Keywords:** funding, guidelines, gynecologic, gynecology, national institutes of health

## Abstract

Objectives

The aim of this study was to report on Research Project Grants (R01) funding in gynecology and to assess its impact on peer-reviewed publications and their incorporation into the American College of Obstetricians and Gynecologists (ACOG) practice guidelines.

Methods

This cross-sectional study was performed using data obtained from the National Institutes of Health (NIH) Research Portfolio Online Report and Tools Expenditure and Results (RePORTER). Publications stemming from R01 grants were determined by extracting the publication list from the individual project data pages. ACOG Practice Bulletins and Committee Opinions related to gynecology were screened. If the principal investigator associated with the R01 grant was referenced in the guidelines, the citation was cross-referenced with the NIH RePORTER website to confirm its origin and incorporation into practice guidelines.

Results

Gynecology received 0.1-0.2% of the over $40 billion total NIH funding. Gynecologic R01 funding increased by 57.7% between 2000 and 2005 and by 55.5% between 2005 and 2010, but it increased minimally between 2010 and 2015 (2.5%). The number of grants followed a similar trend, with a decrease of -36.8% by 2015. Between 2000 and 2010, gynecology had 50% to 70% less funding when compared with obstetrics and urology. During these years, 40% of states did not get a single R01 grant. Since 2005, less than 1% of publications have been referenced in ACOG guidelines and 0.3% or less have been linked to practice recommendations. Out of 5,663 publications, only 11 have been linked with recommendations since the year 2000.

Conclusion

Fewer than 0.3% of R01 grants focused on gynecologic topics, and there was a relative decline in the number of grants and NIH funding allocated to gynecology. The number of publications referenced in ACOG guidelines and linked to recommendations remains minimal, emphasizing a need to fund topics that influence national guidelines.

## Introduction

When measured by disease prevalence and burden, medical research in women’s health is underfunded [1-3]. The National Institutes of Health (NIH) is the dominant funding source for healthcare research in the United States and assigns close to $19 billion in annual grants, with only 1% destined for obstetrics and gynecology [4]. The NIH categorizes grants into different series based on the type of research, the researcher’s career stage, and the grant’s purpose. The R series grants embody the largest and most common category. Among the R series, Research Project Grants (R01) generally offer the most extended funding period for independent researchers. According to previous data, less than 0.5% of R01 focus on obstetrical topics [5], while the funding allocation for gynecology remains undisclosed.

The American College of Obstetricians and Gynecologists (ACOG) publishes Practice Bulletins and Committee Opinions to provide clinical recommendations for healthcare providers in obstetrics and gynecology. These recommendations are evidence-based, patient-centered, and overall best practices that are constantly referenced as standard of care nationwide. Importantly, the influence of NIH funding on ACOG practice guideline recommendations in gynecology remains unknown.

Our study aimed to report on R01 funding in gynecology and evaluate the impact of these grants on peer-reviewed publications and their inclusion into ACOG practice guidelines.

This study was previously presented as an oral abstract at the 53rd AAGL Global Congress on Minimally Invasive Gynecologic Surgery (MIGS), held in New Orleans, Louisiana, in November 2024.

## Materials and methods

A cross-sectional study was conducted using data from the NIH Research Portfolio Online Report and Tools Expenditure and Results (RePORTER) site [6]. R01 grants in gynecology awarded for the first time in 2000, 2005, 2010, and 2015 were included. These grants encompassed various fields, including general gynecology, gynecologic oncology, urogynecology, reproductive endocrinology and infertility, complex family planning, pediatric gynecology, and minimally invasive gynecologic surgery/complex gynecology. To allow for an adequate sample of research output related to these grants, five-year intervals were used in this cross-reference analysis. Projects awarded after 2015 were excluded as these grants support foundational research and publications and practice recommendations resulting from these may not be evident until several years after the grant is awarded.

Peer-reviewed publications originating from R01 grants were determined by extracting the publication list from the individual project data pages. All ACOG Practice Bulletins and Committee Opinions that were available in February 2024 related to gynecology were then screened to determine if the publications were referenced in ACOG guidelines [7,8]. If the R01 principal investigator was referenced, the citation was cross-referenced with the RePORTER website to confirm its origin and incorporation into practice guidelines. The data extraction was independently extracted by three of the authors, and the information was then confirmed by a different author. Total funding for obstetrics and total funding for urology were each extracted separately but using the same methodology from the RePORTER and the Blue Ridge Institute for Medical Research (BIMR) websites.

The study was reviewed by the McGovern Medical School at The University of Texas Health Science Center Institutional Review Board (protocol number HSC-MS-23-0233) and determined exempt. The STROBE guidelines for reporting observational studies were followed [9]. All statistics were performed using Stata V16. The chi-square and Wilcoxon rank sum tests were used for the analysis, and statistical significance was established if the associated value was P<0.5.

## Results

Characteristics of R01 grants are depicted in Table 1.

**Table 1:** Characteristics of Research Project Grants

R01 grants	2000	2005	2000 vs 2005 (% change/p-value)	2010	2005 vs 2010 (% change/p-value)	2015	2010 vs 2015 (% change/ P value)
Total number	27,187	31,271	15.0%	32,182	2.9%	24,966	-22.4%
Total funding ($)	7,511,262,698	10,892,225,932	45.0%	12,249,092,655	12.5%	10,129,566,707	-17.3%
Gynecology topic (%)	24 (0.1)	33 (0.1)	37.5%	57 (0.2)	72.7%	36 (0.1)	-36.8%
Funding for gynecology							
Total funding in gynecology ($) (%)	7,332,588 (0.1)	11,563,612 (0.1)	57.7%	17,977,051 (0.1)	55.5%	18,432,168 (0.2)	2.5%
Mean (SD) funding per grant ($)	305,525 (121,714)	350,412 (193,425)	0.288	315,387 (170,887)	0.375	512,005 (322,637)	0.002
Median (IQR) funding per grant ($)	277,808 (235,099-314,223)	317,366 (268,403-354,800)	0.070	314,450 (225,750-381,799)	0.691	418,359 (316,034-589,918)	0.001
Total gynecology funding ($)/total publications in gynecology	$13,914	$18,297	31.5%	$5,784	-68.4%	$13,204	128.3%
Number of states with R01 (%)	16 (32%)	19 (38%)	0.529	22 (44%)	0.542	17 (34%)	0.305
International Institutions with R01 (%)	0 (0)	0 (0)	x	1 (1.8)	1.000	0 (0)	1.000

A total of 115,606 grants were given between 2000 and 2015, with only 0.1% to 0.2% of those for gynecologic projects. Gynecology received between $7,332,588 (0.1%) and $18,432,168 (0.2%) of the over $40 billion total funding during these four years. Gynecologic R01 funding increased by $4,231,024, which represents 57.7% nominally (44.3% adjusted for inflation) between 2000 and 2005, and by $6,413,439, which represents 55.5% nominally (43.8% adjusted for inflation) between 2005 and 2010. However, funding showed minimal growth of $455,117, which represents 2.5% nominally between 2010 and 2015, translating to a 5.2% real decrease after adjusting for inflation. The number of grants followed a similar trend with 24 grants in 2000, 33 in 2005 (+37.5%), 57 in 2010 (+72.7%), decreasing to 36 in 2015 (-36.8%). Only between 16 (32%) and 22 (44%) states were awarded a grant each year, with 40% of them not receiving a single R01 grant between 2000 and 2015. There was only one international institution awarded a grant throughout that time. Between 2000 and 2010, gynecology had 50% to 70% less funding when compared with obstetrics and urology (Table 2).

**Table 2:** Total funding per specialty

Year	Gynecology	Obstetrics	Urology
2000	$7,332,588	$21,292,084	$12,964,289
2005	$11,563,612	$23,989,876	$20,832,246
2010	$17,977,051	$35,923,163	$35,587,855
2015	$18,432,168	$109,202,694	$43,112,751
Total	$55,305,419	$190,407,817	$112,497,141

In 2015, gynecologic funding was $18,432,168, which represented only 17% of the amount allocated for obstetrics and 42% of the amount allocated for urology. Gynecologic grants funded by the NIH were categorized by topic (Appendix A).

Characteristics of R01 grants and publications in gynecology are shown in Table 3. Publications stemming from R01 grants in gynecology increased from 527 publications in 2000 to 632 in 2005 (+19.9%), 3,108 in 2010 (+391.8%), and decreased to 1,396 in 2015 (-55.1%). The iCite Relative Citation Ratio (RCR) is a metric developed by the NIH to evaluate the influence of scientific publications. While the median number of publications and iCite RCR per grant in gynecology remained relatively stable, there was an increase in the percentage of publications from R01 grants published in major journals between 2010 and 2015 (Table 3).

**Table 3:** Research Project Grants and publications in gynecology

	2000	2005	2000 vs 2005 (% change/p-value)	2010	2005 vs 2010 (% change/p-value)	2015	2010 vs 2015 (% change/p-value)
Publications from R01 in gynecology (number)	527	632	19.9%	3108	391.8%	1396	-55.1%
Number of publications/grants, median (IQR)	9.5 (6-30)	10 (4-20)	0.815	20 (7-38)	0.057	24 (10-60)	0.313
iCite RCR per R01, median (IQR)	1.57 (1.11-2.31)	1.48 (0.88-2.26)	0.605	1.74 (1.18-2.49)	0.672	2.11 (1.19-2.91)	0.263
Publication from R01 in high-impact factor journals, n (%)							
JAMA, Lancet, NEJM (%)	4 (0.8)	2 (0.3)	0.420	15 (0.5)	0.754	37 (2.7)	<0.001
AJOG, BJOG, Obtst Gynecol (%)	150 (28.5)	79 (12.5)	<0.001	155 (5.0)	<0.001	176 (12.6)	<0.001
Others (%)	373 (70.8)	551 (87.2)	<0.001	2938 (94.5)	<0.001	1183 (84.7)	<0.001
ACOG guidelines							
Publications ref (%)	9 (1.7)	4 (0.6)	0.098	11 (0.4)	0.301	10 (0.7)	0.103
Publications linked with Rec (%)	2 (0.4)	2 (0.3)	1.000	3 (0.1)	0.201	4 (0.3)	0.213

Since the year 2000, of the 5,663 publications from R01 grants, 34 (0.6%) have been referenced in ACOG practice guidelines, and only 11 (0.2%) have been linked to ACOG guideline recommendations. In this period of time, $55,305,419 was allocated to gynecologic project grants, resulting in these 11 publications that led to recommendations (Table 4).

**Table 4:** Publications linked to recommendations

ACOG Gynecologic Practice Bulletin Publication	Principal investigator	Year
Std Risks and Latino Adolescents' Sexual Networks	Padian, Nancy S	2000
Treatment Evaluation in Premenstrual Syndrome Patients	Freeman, Ellen W	2000
Vulvar Vestibulitis Trial: Desipramine-Lidocaine	Foster, David Charles	2005
Mechanisms of Anterior Vaginal Wall Support Failure	Delancey, John OL	2005
Risk Factors as Predictors of Ectopic Pregnancy	Barnhart, Kurt T	2010
Mechanisms of Anterior Vaginal Wall Support Failure	Delancey, John OL	2010
Ec Method: Determinants for Copper IUD Use and Future Unintended Pregnancy	Turok, David	2010
Pelvic Floor Disorders Network Duke University Clinical Site	Visco Anthony G	2015
Biomarkers of Infertility	Steiner, Anne Zweifel	2015
Using Comparative Effectiveness Analyses to Optimize Cervical Cancer Screening	Sawaya, George F	2015
Characterization & Prevention of Chemotherapy-Induced Damage to Ovarian Reserve	Oktay, Kutluk H	2015

## Discussion

Our study highlights a relative decline in both the number of R01 grants and NIH funding allocated to gynecologic topics. This trend is particularly concerning given the already minimal proportion of R01 grants designated for this field. Notably, only 34 (0.6%) publications stemming from these grants have been cited by ACOG and just 11 (0.2%) have resulted in changes to clinical practice guidelines. The limited number of publications referenced in ACOG guidelines underscores the pressing need to prioritize funding on topics that directly influence national guidelines.

Women’s reproductive health research is critically underfunded. Our analysis revealed that only 0.1%-0.2% of the total NIH funding was designated to gynecology. This underinvestment likely contributes to the poor state of women’s healthcare in the United States. As an example, research funding for gynecologic cancers is disproportionately lower compared with other cancers. Between 2007 and 2014, the average NIH funding for cervical, uterine, and ovarian cancers was significantly less than that for other malignancies, with uterine cancer receiving $57,000, ovarian cancer $97,000, and cervical cancer $87,000 per person-years life lost per 100 new cases. In contrast, prostate cancer received $1.81 million per person-years life lost per 100 new cases [10]. Additionally, for procedures of similar complexity, insurers commonly reimburse at lower rates for services provided to women compared to those provided to men [11]. These funding disparities are consistent with our findings, which show that R01 funding for gynecology was substantially less than that for urology between 2000 and 2015.

It is noteworthy that within the field of women’s health, the majority of federal grant funding has been allocated to obstetric-related research. Since 2000, funding for gynecology has consistently been less than 50% of that allocated to obstetrics. In gynecology, family planning is significantly undersupported, with only 23 (9%) of the 244 gynecology-related grants in 2023 addressing this topic [12]. Similarly, despite the high prevalence of endometriosis among women, only 41 (16%) grants in 2023 focused on this condition [12]. Endometriosis received $7 million in NIH funding in 2018, placing it at the very bottom of the NIH’s per disease/research-area funding list [13]. While this has improved significantly, with $29 million allocated to endometriosis research in 2023, this amount remains insufficient given the high prevalence, underdiagnosis, and overall poor understanding of its causal mechanisms and treatment. To put this disparity into perspective, endometriosis affects at least 10% of reproductive-age women, while inflammatory bowel diseases (IBDs), such as Chron’s disease or ulcerative colitis, affect approximately 1.3% of the population. Yet, the $29 million allocated to endometriosis research in 2023 represents only 14% of the $199 million allocated to IBD [12]. Following a period of growth from 2000 to 2010, the decline in gynecologic R01 grants, publications, and funding between 2010 and 2015 is equally concerning.

The NIH Revitalization Act of 1993 promoted inclusivity in medical research [14]. It is striking that 20 (40%) states never received R01 funding in gynecology during the study period. This disparity is likely multifactorial, with contributing factors including institutional research capacity, the geographic distribution of academic medical centers, and the competitive nature of NIH funding. Efforts should be taken to prioritize inclusivity and ensure the research participation of women from diverse ethnic, socio-economic, and geographical backgrounds. Equally important is the need to foster career pathways for physician-scientists across the nation. This cohort is a driving force behind practice-changing research, yet within women’s health, physician-scientists are relatively uncommon and often face significant barriers [15,16].

A key strength of our study lies in its comprehensive analysis, which extends beyond NIH funding in gynecology to include the number of grants, funding trends, resulting publications, and the overall research impact. Additionally, our systematic review of all Practice Bulletins and Committee Opinions offers valuable insights into the role of NIH funding in shaping practice guidelines. This is particularly relevant, as these guidelines are widely regarded as the standard of care nationwide. Our study has limitations that need to be acknowledged. First, R01 can provide mechanistic insights or proof of concept data that may lead to additional research funded through alternative mechanisms that were not accounted for. Second, other federal grants including NIH grant types beyond R01 may have contributed to the development of ACOG guidelines but were not included in our study. Finally, we recognize that ACOG guidelines do not encompass the full scope of gynecologic subspecialty care. As such, many R01-funded projects may influence recommendations from gynecologic subspecialty societies that were not considered in this analysis.

## Conclusions

The impact of R01 publications on clinical practice appears minimal, with only 11 publications (0.2%) resulting in practice recommendations. For one publication to be linked with ACOG practice guidelines, it took an average of $5,027,656. These findings can serve as a powerful tool for advocating for increased and targeted funding for gynecologic research in the future.

## Appendices

Appendix A

**Table 5:** Total funding in gynecology per topic GTN, gestational trophoblastic neoplasia; PCOS, polycystic ovary syndrome

Topic per year	Number of Grants	Funding (%)
2000		
Pelvic floor disorders	7	$1,874,023 (25.6)
Sexual health	3	$1,176,160 (16)
Menopause	3	$969,964 (13.2)
Mental health in gynecology	1	$759,801 (10.4)
Ectopic pregnancy	2	$577,721 (7.9)
Ovarian cancer	2	$452,616 (6.2)
Pelvic pain	1	$314,000 (4.2)
Premenstrual syndrome	1	$300,202 (4)
Endometriosis	1	$263,695 (3.5)
PCOS	`1	$253,902 (3.4)
Urinary incontinence	1	$195,500 (2.6)
Surgical infection prevention	1	$195,004 (2.6)
Total	24	$7,332,588
2005		
Menopause	3	$2,185,269 (18.9)
Contraception	4	$1,343,567 (11.6)
Fibroids	4	$1,103,211 (9.5)
Endometriosis	3	$1,064,636 (9.2)
Pelvic pain	3	$919,837 (7.9)
Pelvic floor disorders	3	$775,913 (6.7)
Urinary incontinence	2	$668,441 (5.7)
Endometrial cancer	2	$633,441 (5.4)
Cervical cancer	2	$608,828 (5.2)
Hysterectomy	1	$581,972 (5)
Ovarian cancer	2	$500,754 (4.3)
Premenstrual syndrome	1	$356,625 (3.1)
Abnormal uterine bleeding	1	$285,400 (2.4)
GTN	1	$270,000 (2.3)
PCOS	1	$265,718 (2.2)
Total	33	$11,563,612
2010		
Ovarian cancer	8	$2,667,788 (14.8)
Endometriosis	8	$2,376,591 (13.2)
Pelvic floor disorders	8	$2,168,269 (12)
Sexual health	4	$1,865,661 (10.4)
Fibroids	4	$1,448,545 (8)
Cervical cancer	3	$1,116,438 (6.2)
Menopause	3	$1,049,277 (5.8)
PCOS	4	$1,045,097 (5.8)
Gynecologic cancer	2	$906.075 (5)
Contraception	3	$818,050 (4.5)
Ectopic pregnancy	1	$603,532 (3.3)
Endometrial Cancer	2	$581,982 (3.2)
Ovarian physiology	2	$543,442 (3)
Reproductive health	2	$442,289 (2.5)
Urinary incontinence	2	$314,272 (1.7)
Uterine physiology	1	$29,743 (0.2)
Total	57	$17,977,051
2015		
Pelvic floor disorders	7	$4,354,170 (23.6)
Ovarian cancer	8	$3,123,057 (16.9)
Reproductive medicine	3	$3,014,521 (16.4)
Contraception	5	$2,480,683 (13.5)
Endometriosis	3	$1,148,548 (6.2)
Cervical cancer	2	$1,098,640 (5.9)
Fibroids	2	$989,405 (5.4)
Endometrial cancer	3	$925,311 (5)
Ectopic pregnancy	1	$661,771 (3.6)
Infertility	1	$443,867 (2.4)
Urinary incontinence	1	$192,195 (1)
Total	36	$18,432,168
